# Health care providers’ compliance with the notifiable diseases surveillance system in South Africa

**DOI:** 10.1371/journal.pone.0195194

**Published:** 2018-04-09

**Authors:** Frew Gerald Benson, Jonathan Levin, Laetitia Charmaine Rispel

**Affiliations:** 1 Gauteng Department of Health, Rahima Moosa Hospital, Newclare, Johannesburg, South Africa; 2 School of Public Health, Faculty of Health Sciences, University of the Witwatersrand, Parktown, Johannesburg, South Africa; 3 Centre for Health Policy, Department of Science and Technology/National Research Foundation, SARChI Chair on the Health Workforce, School of Public Health, Faculty of Health Sciences, University of the Witwatersrand, Parktown, Johannesburg, South Africa; University of Melbourne, AUSTRALIA

## Abstract

**Background:**

The optimal performance of a notifiable disease surveillance system (NDSS) is dependent on health care provider (HCP) compliance with communicable disease notification. HCP compliance ensures appropriate investigation and control measures by relevant health care authorities. This study examines the compliance of HCPs with the NDSS in South Africa and factors associated with their compliance.

**Methods:**

A cross-sectional survey was carried out in three randomly selected provinces. We stratified by type of facility, and recruited clusters of HCPs on survey day to participate. All consenting HCPs in the randomly selected health care facilities on the day of the survey, completed a questionnaire that elicited information on socio-demographic characteristics and notification practices. The data were analysed using STATA^®^ 14, using the identifiers for stratum and cluster as well as the calculated sampling weights.

**Results:**

The study found that 58% of 919 HCPs diagnosed a notifiable disease in the year preceding the survey. The majority of these professionals (92%) indicated that they had reported the disease, but only 51% of those notified the disease/s correctly to the Department of Health. Paediatricians were less likely to notify correctly (OR 0.01, 95% CI 0.00–0.12, p = 0.001). The factors that influenced notification were HCPs perceptions of workload (OR 0.84, 95% CI 0.70–0.99, p = 0.043) and that notification data are not useful (OR 0.84, 95% CI 0.71–0.99, p = 0.040). The study found no association between correct notification and HCPs’ willingness to notify, experience or training on the NDSS, understanding of the purpose of the NDSS, knowledge of what to notify, or perception of feedback given.

**Conclusions:**

The compliance of HCPs in South Africa with the NDSS is suboptimal. In light of the important role of HCPs in the effective functioning of the NDSS, information on NDSS usefulness and guidelines on correct notification procedures are needed to increase their compliance.

## Introduction

The 2014 to 2016 Ebola Virus Disease outbreak [1], the 2016 Zika virus [2] and yellow fever outbreaks [3] underscore the need for effective country-based notifiable diseases surveillance systems (NDSS). An effective NDSS enables a country to deal with outbreaks of emerging and re-emerging communicable diseases at source and to prevent their spread within and beyond its borders.

Health care providers (HCPs), defined as medical doctors and professional nurses, are critical to strong, resilient health systems, [4] and are at the coalface of service delivery, responsible for the diagnosis and effective management of infectious diseases. The optimal performance of the NDSS is dependent on HCP compliance with communicable disease notification. Their compliance ensures appropriate investigation and control measures by relevant health care authorities. Furthermore, compliance with the NDSS facilitates uniformity in morbidity and mortality reporting that allows for comparisons within and among countries. However, differences in context, background, healthcare systems and resource availability complicate comparisons among countries. Nonetheless, many countries around the world have made it mandatory for HCPs to notify certain notifiable diseases upon clinical suspicion and/or laboratory confirmation [5–7].

Despite this legal obligation, underreporting of notifiable diseases is a common problem for passive surveillance systems in all countries, regardless of income [8–32]. Many high-income countries (HICs) [8, 15, 18, 33] have introduced measures to make it compulsory for laboratories to notify communicable diseases and have dual reporting systems in order to overcome the problem of under-reporting. However, the strong laboratory networks in these HICs proved effective in improving the functioning of the NDSS. In contrast, in low- and middle-income countries (LMICs), a 2009 review of NDSS evaluations found that resource constraints and health infrastructure challenges contributed to sub-optimal NDSS functioning [34]. In these countries, particularly in Africa, laboratory networks are relatively weak and the reliance on HCPs to notify diseases is therefore stronger. Hence, there is a need in these countries to assess the level of compliance amongst HCPs periodically and to take steps to identify and address the factors which cause non-compliance or under-reporting.

In South Africa, the NDSS is a paper-based system that tracks 33 medical conditions. Existing legislation obliges all HCPs to notify these conditions to their local authority, which in turn reports it to the district, district to province, and province to the national department of health (DOH) [7, 35]. There are no legal provisions for laboratories to notify any communicable disease in the country.

In South Africa, there have only been a few evaluations of HCPs compliance since the inception of the NDSS in the 1970s [36–38]. These include studies on reporting of hepatitis B for the period 1985 to 1988 [39], rheumatic fever for the period 1990–2004, and notifications amongst private general practitioners (GPs) in Gauteng province in 2006. A limitation of these previous studies is that they precede the implementation of the International Health Regulation (IHR) and are focused on limited geographical settings or diseases and hence cannot be extrapolated to the entire NDSS. There is a dearth of information on factors associated with HCPs compliance with the NDSS. Furthermore, the South African health system has undergone and continues to undergo major changes, and is inter-connected with a global world. Information on HCP compliance is important for shaping NDSS reforms, ensuring effective disease response and compliance with the IHR, and monitoring trends and/or benchmarking the performance of HCPs [4]. In light of this, and the dearth of empirical information, the objectives of this study were to determine HCPs’ compliance with the NDSS in South Africa; and to determine the factors associated with their compliance. The study is part of broader doctoral study to evaluate the performance of the NDSS in South Africa.

## Methods

### Study setting

The study was conducted in three of the nine South African provinces, one of each representing the urban, rural and the mixed urban-rural groups of provinces.

### Study population

The study population consisted of all HCPs, specifically doctors and professional nurses (with four years of training) involved with communicable diseases, working in the public and private health care sectors at primary health care (PHC) and hospital levels.

Enrolled and student nurses were excluded from the study. There were 49,260 eligible HCPs working in the selected facilities (see sampling design).

### Sample size

The sample size was estimated using Epi Info statistical software, version 7.2, for population surveys, assuming: a) the acceptable margin of error as ± 5%; b) approximately 40% would notify correctly (estimated from a previous Gauteng study [38]); and c) design effect of at most 2.5 to account for clustering effects (this is conservative). This required an overall sample size of 936 HCPs. To allow for a non-response of 10% we targeted 1050 HCPs. This number was 2.1% of the total eligible HCPs in the study population.

### Sampling design

Three of the nine provinces were selected randomly, and the final sample consisted of the three provinces of Gauteng (urban), KwaZulu-Natal (mixed urban-rural) and Limpopo (rural). We then stratified by type of health facilities, namely central /tertiary hospitals, regional hospitals, district hospitals, primary health care facility (which included community health centres (CHCs) and clinics), private hospitals and private general practitioners (GPs). Satellite and mobile clinics (as they operate only for a few hours per week and therefore see a very limited number of patients), as well as private hospitals with fewer than 100 beds (which have limited scope and practices, and do not have the targeted health disciplines that deal with the NDSS) were excluded from the study. The target sample size for each province was chosen proportional to the number of HCPs in that province. In order to select the number of facilities of each type to be sampled in each province, we assumed an average number of HCPs for that facility (e,g. we assumed that each clinic would contain 7 HCPs on the day of the survey); a pre-specified number was chosen to lead to an overall sample of 1050 HCPs. This allowed us to select a sample of facilities randomly in each province. In each sampled facility, all nurses and doctors who worked in specific divisions (internal medicine, out-patients, medical casualty, critical care, paediatrics and infection control units) were targeted on the survey day. In PHC, all nurses and doctors were requested to participate in the study. We also randomly selected 70 general practitioners (GPs) in private practice as part of the “private sector facilities” (20 in Gauteng province, 20 in Limpopo and 30 in KwaZulu-Natal) using an electronic database of private GPs. Sampling weights were calculated based on the total number of HCPs in each stratum.

### Measurement and data collection

We could not find a standardised tool to measure HCP compliance with the national NDSS. We designed a self-administered questionnaire in English, the official business language of South Africa (S1 File). The questions focussed on socio-demographics, participant knowledge, attitudes and practices to disease notification and factors influencing compliance with notification reported in the literature. We defined “correct notification” as notification to the local, provincial or national DOH. An assessment of the quality of the information they provided to the DOH did not form part of this study and is therefore not included in the definition of “correct notification”. We calculated Cronbach’s alpha coefficients to determine reliability and coherence between items—they ranged from 0.82 to 0.97, indicating high reliability and inter-item correlation. We piloted the questionnaire among 12 HCPs similar to the study population prior to data collection to determine clarity of questions and time taken for administration, and no changes were deemed necessary.

The survey was conducted from 22 May to 19 June 2015. Twelve professional nurses were recruited as field workers and trained to assist with data collection in the selected facilities.

On the day of the survey, all eligible study participants were given an information sheet and requested to participate in the study on a voluntary basis.

We double captured data into the web-based Research Electronic Data Capture (REDCap), programme hosted at the University of Witwatersrand in Johannesburg [40].

### Data analysis

We exported the data into STATA^®^ 14 for cleaning and analysis. To specify the survey design, we used the stratum (combination of province and type of facility), the cluster (i.e. facility) and calculated sampling weights based on the number of doctors and nurses in each stratum. All analyses were appropriate for survey data using the identifiers for stratum and cluster as well as the calculated sampling weights.

We computed frequency and summary tables to describe participants’ age, gender, location, professional category, experience and training on the NDSS. We summarized categorical variables in tables showing frequency and weighted percentages of each category. We summarized numerical variables using medians (inter-quartile ranges).

We analysed the number and weighted percentage of HCPs who diagnosed and notified notifiable diseases in the preceding year. We used the Rao-Scott correction to the Chi-square test [41] to compare the practices of public and private HCPs. The outcome variable was correct notification of relevant disease/s. We determined whether participants’ age, gender, experience, training, professional category, ownership (public or private) and place of employment, workload, possession of notification forms, knowledge of the NDSS were associated with correct notification (to the DOH) using robust survey logistic regression. We calculated odds ratios (OR), 95% confidence intervals (95% CI) and p-values. P-values of less than 0.05 were considered to be statistically significant.

### Ethics approval and consent to participate

The Human Research Ethics Committee (Medical) of the University of the Witwatersrand in Johannesburg provided approval for the study (Clearance certificate M140624). All participants provided written informed consent, no personal identifiers were collected and data are reported in aggregate.

## Results

### Socio-demographic factors of health care providers

Of the total of 1,050 HCPs that were targeted, we enrolled a total of 942 participants (response rate 90%). After excluding those who did not give consent (n = 5), were enrolled nurses (n = 10), or did not state their professional category (n = 8), the final sample size was 919. The majority of participants (81%) were in the public health sector and professional nurses (76%).

The median age of the HCPs was 41 years, inter-quartile range (IQR) 33 to 50 years. In the public sector, the median age of doctors was 37 years (IQR 30–44). The majority of all participants were female (81%). In the private sector, the majority of doctors were male (72%), compared to the public sector in which the majority of doctors were female (55%). In the public health sector, 39% of doctors were from regional hospitals, and 41% of nurses from PHC facilities. In the private sector, 59% of nurses reported that they were trained in the NDSS, while 41% of public sector nurses reported NDSS training. The median number of years of experience in the NDSS was 4 years (IQR 0–10). The experience of private sector doctors, 10 years (IQR 3–25), was higher than public sector doctors, 6 years (IQR 0–15) (Table 1).

**Table 1:** Socio-demographic factors of health care providers 2015.

Variable	Public Sector N (%)		Private Sector N (%)		Total
	Medical Doctors	Professional Nurses	Medical Doctors	Professional Nurses	n = 919
**Province**					
Gauteng	91 (46)	178 (43)	14 (40)	91 (42)	374 (43)
KwaZulu-Natal	68 (30)	188 (35)	17 (48)	98 (45)	371 (36)
Limpopo	41 (24)	96 (21)	8 (11)	29 (13)	174 (20)
Total	200 (100)	462 (100)	39 (100)	218 (100)	919 (100)
**Type of facility**					
Central/Tertiary	76 (7)	52 (1)			128 (3)
Regional Hospital	68 (39)	78 (13)			146 (19)
District Hospital	34 (36)	68 (23)			102 (26)
PHC	22 (11)	264 (41)			286 (34)
Private Hospitals			11 (4)	218 (23)	229 (18)
Private GP practice			28 (3)	0 (0)	28 (1)
Total	200 (94)	462 (77)	39 (6)	218 (23)	919 (100)
**Age Median (IQR)**	37 (30–44)	43 (34–52)	41 (36–52)	41 (34–49)	41 (33–50)
**Age** (years)					
20–30	55 (30)	71 (15)	1 (2)	28 (14)	155 (18)
31–40	64 (33)	103 (27)	17 (46)	61 (33)	245 (30)
41–50	38 (24)	121 (30)	8 (22)	66 (35)	233 (29)
51–60	12 (6)	90 (23)	7 (25)	27 (14)	136 (18)
61–70	11 (7)	15 (4)	3 (5)	5 (3)	34 (5)
Total	180 (100)	400 (100)	36 (100)	187 (100)	803 (100)
**Gender**					
Female	104 (55)	415 (89)	8 (28)	196 (90)	723 (81)
Male	96 (45)	43 (11)	31 (72)	21 (10)	191 (19)
Total	200 (100)	458 (100)	39 (100)	217 (100)	914 (100)
**Nurse Category**					
PHC Trained		153 (31)		19 (9)	172 (26)
General Nurse		217 (46)		102 (47)	319 (46)
Trauma and Casualty		2 (1)		17 (8)	19 (2)
ICU and High Care		11 (2)		27 (12)	38 (4)
Midwifery		14 (5)		7 (3)	21 (4)
Theatre		1 (0)		8 (4)	9 (1)
Infection Prevention		29 (7)		22 (10)	51 (8)
Paediatric		35 (9)		16 (7)	51 (8)
Total		462 (100)		218 (100)	680 (100)
**Doctor Category**					
Intern	17 (7)		0 (0)		17 (6)
Medical Officer	92 (63)		2 (7)		94 (60)
Private GP	13 (7)		27 (43)		40 (9)
Registrar	23 (4)		0 (0)		23 (4)
Specialist Physician	19 (4)		6 (29)		25 (5)
Paediatrician	29 (14)		2 (11)		31 (14)
Other Specialists	7 (2)		2 (11)		9 (3)
Total	200 (100)		39 (100)		239 (100)
**Training in NDSS**	66 (35)	180 (41)	16 (44)	126 (59)	388 (43)
**Formal training in Epidemiology**	52 (27)	78 (16)	11 (24)	51 (24)	192 (20)
**Experience in the NDSS—years**					
Median (IQR)	6 (0–15)	4 (0–10)	10 (3–25)	4 (0–10)	4 (0–10)

**Weighted percentages are presented**. NDSS = Notifiable Diseases Surveillance System. GP = General Practitioner. ICU = Intensive Care Unit. PHC = Primary Health Care. IQR = Inter quartile range

### Diagnosis of notifiable diseases

More than half of the HCPs (58%), reported that they have diagnosed a notifiable disease in the year preceding the survey, with a significantly higher percentage in the public sector (62%), compared to 43% in the private sector (p = 0.001). A significantly higher percentage of public sector nurses in KwaZulu-Natal province (57%) reported diagnosis of a notifiable disease, compared to private sector nurses (41%, p = 0.019). In the public sector, 94% of doctors, reported diagnosis of a notifiable disease, compared to 89% in the private sector.

**Fig 1:**
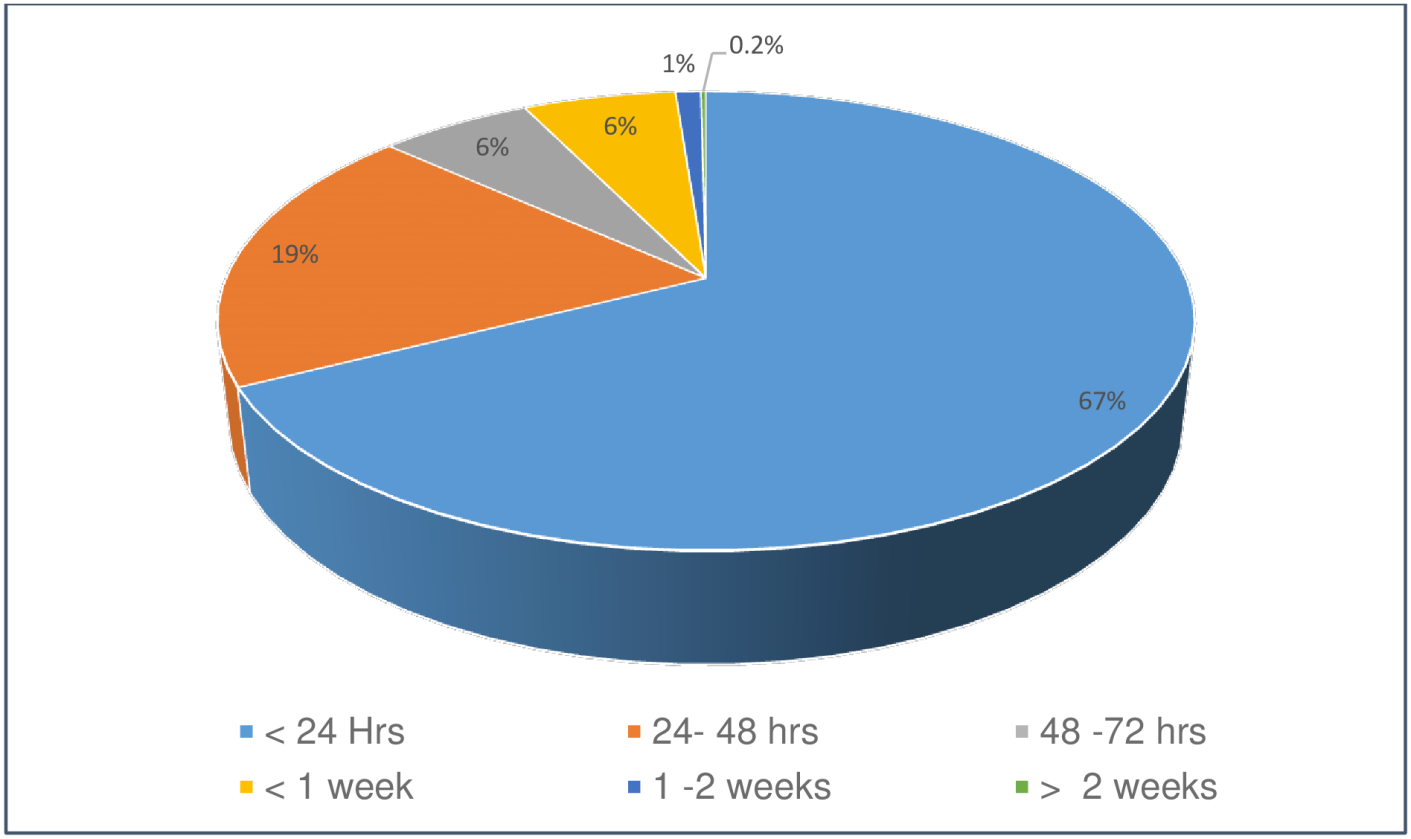
Timeframe after diagnosis of a notifiable communicable disease in the preceding year that health care providers reported they notified the disease, South Africa, 2015 (weighted %).

In both the public and private sectors, 92% of all study participants who have diagnosed a notifiable disease(s) indicated that they reported the notifiable disease(s). Public sector doctors in KwaZulu-Natal and private sector doctors in Limpopo notified lower percentages of cases (80% and 75% respectively) compared to other HCPs (Table 2). Two thirds (67%) of HCPs who indicated that they notified in the preceding year, reported that they did so within 24 hours of diagnosis; only 1% reported that they did so after more than 1 week (Fig 1).

**Table 2:** Health care providers who: (a) have diagnosed, and (b) notified a notifiable communicable disease in the preceding year, South Africa, by sector, professional category and province, 2015.

**a) Health Care Providers who have diagnosed a notifiable disease in the preceding year**									
**i) By Sector**									
**Sector**	**No**	**Yes**	**Unsure**	**Total**					**P-value**^c^
Public	231 (37)	397 (62)	11 (1)	639 (100)					
Private	128 (56)	113 (43)	3 (1)	244 (100)					0.001*
Total	359 (41)	510 (58)	14 (1)	883 (100)					
**ii) Doctors by Province**									
**Province**	**Public Sector**				**Private Sector**				
	**No**	**Yes**	**Unsure**	**Total**	**No**	**Yes**	**Unsure**	**Total**	
Gauteng	9 (8)	81 (92)	0 (0)	90 (46)	2 (17)	12 (83)	0 (0)	14 (40)	0.094
KwaZulu-Natal	6 (6)	61 (93)	1 (1)	68 (30)	3 (9)	14 (91)	0 (0)	17 (48)	0.715
Limpopo	2 (1)	38 (98)	1 (2)	40 (24)	0 (0)	8 (100)	0 (0)	8 (11)	0.902
Total	17 (6)	180 (94)	2 (1)	197 (100)	5 (11)	34 (89)	0 (0)	39 (100)	0.199
**iii) Professional Nurses by Province**									
Gauteng	97 (59)	59 (40)	3 (1)	159 (43)	53 (65)	28 (34)	1 (1)	82 (42)	0.512
KwaZulu-Natal	84 (42)	96 (57)	5 (2)	185 (35)	57 (59)	39 (41)	0 (0)	96 (45)	0.019*
Limpopo	33 (43)	62 (54)	1 (1)	96 (21)	13 (48)	12 (44)	2 (7)	27 (13)	0.502
Total	214 (49)	217 (49)	9 (2)	440 (100)	123 (60)	79 (39)	3 (1)	205 (100)	0.073
**b) Health Care Providers who have notified the diagnosed disease(s) in the preceding year**									
**i) By Sector**									
**Sector**	**No**	**Yes**	**Unsure**	**Total**					
Public	26 (5)	358 (92)	11 (3)	395 (100)					
Private	11 (6)	100 (92)	2 (1)	113 (100)					0.602
Total	37 (6)	458 (92)	13 (3)	508 (100)					
**ii) Doctors by Province**									
**Province**	**Public Sector**				**Private Sector**				
	**No**	**Yes**	**Unsure**	**Total**	**No**	**Yes**	**Unsure**	**Total**	
Gauteng	7 (7)	73 (93)	1 (0.1)	81 (46)	1 (4)	11 (96)	0 (0)	12 (40)	0.720
KwaZulu-Natal	8 (11)	48 (80)	5 (9)	61 (30)	3 (10)	10 (87)	1 (3)	14 (48)	0.906
Limpopo	2 (3)	35 (94)	1 (2)	38 (24)	2 (25)	6 (75)	0 (0)	8 (11)	0.115
Total	17 (7)	156 (89)	7 (4)	180 (100)	6 (10)	27 (89)	1 (2)	34 (100)	0.736
**iii) Professional Nurses by Province**									
Gauteng	4 (6)	53 (91)	1 (3)	58 (43)	3 (11)	25 (89)	0 (0)	28 (42)	0.189
KwaZulu-Natal	3 (2)	91 (97)	1 (1)	95 (35)	2 (5)	36 (92)	1 (3)	39 (45)	0.368
Limpopo	2 (4)	58 (93)	2 (4)	62 (21)	0 (0)	12 (100)	0 (0)	12 (13)	0.688
Total	9 (4)	202 (94)	4 (2)	215 (100)	5 (6)	73 (92)	1 (1)	79 (100)	0.333

**Weighted** %. "Unsure" was regarded as missing data in the calculation of P-values.

* Significant P-value

^c)^ Test specific P-values are from the Rao-Scott correction to the Chi-square Test [41]

### Correct notification of diseases

**Fig 2:**
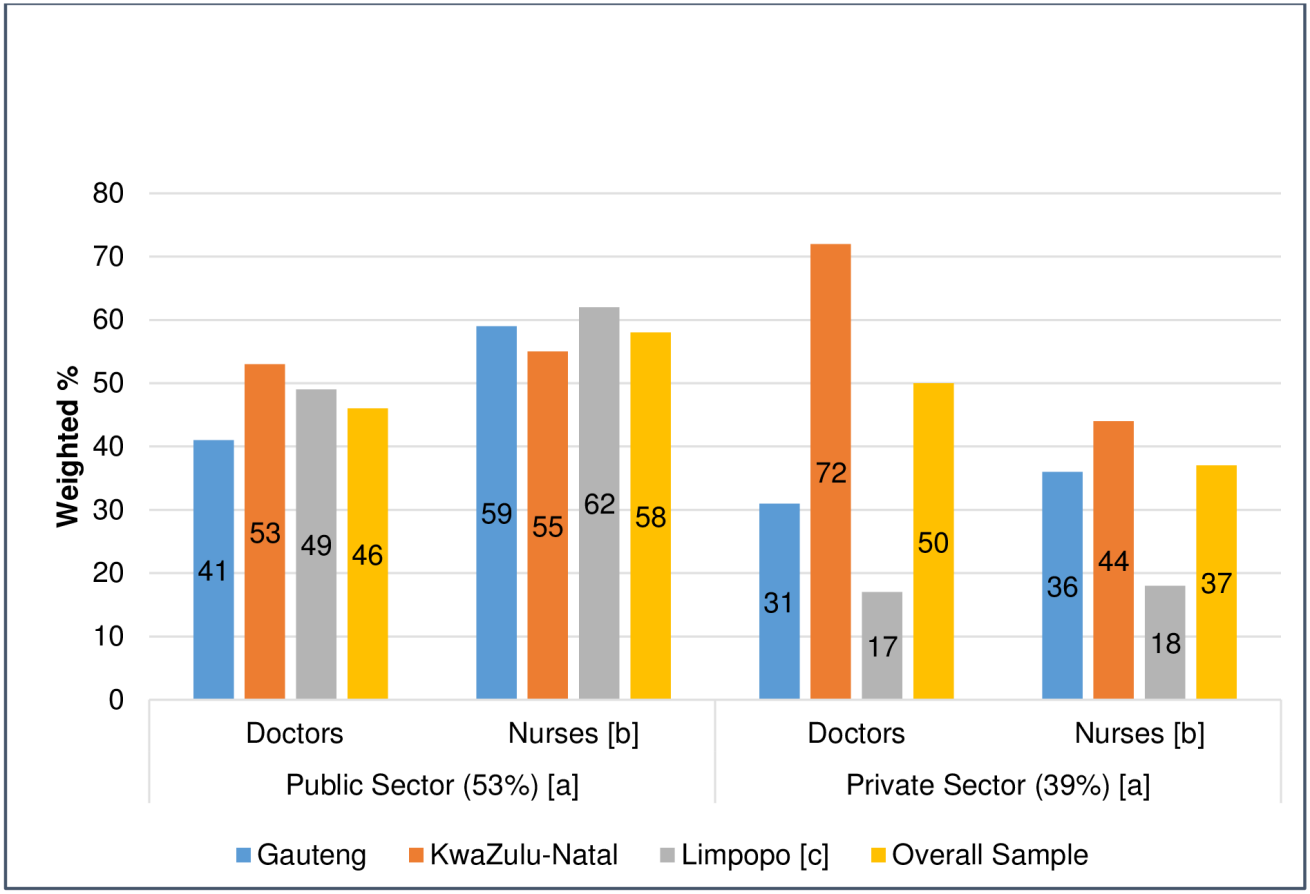
Reported correct notification by health care providers in South Africa, 2015. P-values—Roa Scott correction to Chi-square Test (Public vs Private). a. 0.091. b. 0.042*. c. 0.001* and 0.006*.

Of the HCPs who indicated they reported diseases, only 217 (51%) indicated that they notified the case(s) to the DOH, while 34% of HCPs reported notifiable disease(s) to institutional infection control nurses; these categories where mutually exclusive—the few who indicated they reported to the department and others, were taken as reporting to the department (Table 3). Fewer HCPs in the private sector notified cases correctly, but this was not statistically significant (p = 0.091). Professional nurses in the public sector (58%) were more likely than those in the private sector (37%) to report, correct notification (p = 0.042). In Limpopo province, private sector HCPs reported significantly lower correct notifications (Fig 2).

**Table 3:** Reporting authority of notifiable communicable diseases by sector, province and professional category, South Africa.

**a) Who reported to by sector and province**							
**Who reported to**	**Public Sector N (%)**			**Private Sector N (%)**			**Total**
	**Gauteng**	**KwaZulu-Natal**	**Limpopo**	**Gauteng**	**KwaZulu-Natal**	**Limpopo**	
Department	63 (49)	70 (55)	47 (57)	13 (35)	21 (49)	3 (18)	217 (51)
Infection control	41 (39)	48 (30)	26 (28)	14 (45)	14 (34)	8 (58)	151 (34)
Local clinic or hospital	6 (3)	7 (5)	1 (1)	4 (3)	4 (3)	2 (16)	24 (4)
Nurse in charge or Doctor	10 (8)	11 (6)	13 (13)	4 (13)	3 (7)	3 (6)	44 (9)
Patient and other	1 (0.1)	2 (4)	1 (1)	1 (3)	3 (7)	1 (2)	9 (2)
Total	123 (100)	138 (100)	87 (100)	36 (100)	45 (100)	17 (100)	445 (100)
**b) Who reported to by sector and professional category**							
**Who reported to**	**Public Sector N (%)**			**Private Sector N (%)**			**Total**
	**Medical Doctors**	**Professional Nurses**	**Public Total**	**Medical Doctors**	**Professional Nurses**	**Private Total**	
Department	70 (46)	110 (58)	180 (53)	11 (50)	26 (37)	37 (39)	217 (51)
Infection control	58 (37)	57 (30)	115 (33)	5 (29)	31 (44)	36 (42)	151 (34)
Local clinic or hospital	6 (2)	8 (5)	14 (4)	8 (14)	2 (3)	10 (5)	24 (4)
Nurse in charge or Doctor	21 (15)	13 (4)	34 (9)	3 (5)	7 (10)	10 (9)	44 (9)
Patient and other	1 (0.1)	3 (3)	4 ((2)	1 (2)	4 (6)	5 (5)	9 (2)
Total	156 (100)	191(100)	347 (100)	28 (100)	70 (100)	98 (100)	445 (100)

**Weighted percentages are presented**. All categories where mutually exclusive—the few who indicated they reported to the department and others, were taken as reporting to the department

### Factors associated with correct notification

Unadjusted logistic regression analysis showed that general and critical care nurses were significantly less likely to notify correctly and that HCPs in PHC facilities were more likely to report correct notification. Other factors significant in unadjusted analysis were the possession of notification forms, knowledge of the notification process and the perception of ease of the notification process. All of the aforementioned factors were not significant in multivariable analysis.

Only 15% of paediatricians notified correctly, with multivariable analysis showing that paediatricians were less likely to notify a notifiable disease correctly (OR 0.01, 95% CI 0.00–0.12, p = 0.001) (Table 4). It also showed that HCPs perceptions of their workload (OR 0.84, 95% CI 0.70–0.99, p = 0.043) and their belief that notification data are not used for outbreak response (OR 0.84, 95% CI 0.71–0.99, p = 0.040), had a significant impact on them notifying diseases. The number of HCP patient consultations per day had no significant association with correct notification (OR 1.00, 95% CI 1.00–1.01, p = 0.578).

**Table 4:** Factors associated with health care providers notifying a notifiable communicable disease correctly in the preceding year, South Africa, 2015.

Factor	Unadjusted Logistic regression			Multiple Logistic regression		
	Odds Ratio	95% CI	P-value	Odds Ratio	95% C I	P-value
Age	1.02	1.00–1.04	0.063			
Gender	0.97	0.51–1.82	0.912			
Years’ experience	1.02	0.99–1.05	0.261			
Professional Category	1.36	0.71–2.59	0.345			
Paediatrician	0.07	0.01–0.59	0.017*	0.01	0.00–0.12	0.001*
General nurse	0.44	0.24–0.89	0.012*	1.29	0.57–2.91	0.522
Critical Care Nurse	0.01	0.00–0.12	0.001*	0.08	0.01–1.03	0.053
Infection Prevention Nurse	1.68	0.41–6.90	0.457			
Type of facility	1.3788	1.14–1.67	0.002*	1.38	0.41–4.59	0.583
Primary health facility	3.54	1.62–7.76	0.002*	3.80	0.01–1233.74	0.637
Sector Employed	0.57	0.30–1.11	0.094			
Knowledge of notification process	1.07	1.00–1.14	0.037*	0.99	0.85–1.14	0.843
Willingness to notify	1.00	0.90–1.12	0.963			
Not easy to comply	0.85	0.74–0.98	0.025 *	0.94	0.80–1.11	0.473
Training on NDSS	1.16	0.83–1.61	0.383			
Formal training Epidemiology	1.45	0.84–2.50	0.176			
Understanding the purpose of NDSS	0.97	0.88–1.14	0.736			
Knowledge on what to notify immediately	1.07	0.97–1.18	0.153			
Knowledge on what to notify in 24hrs	1.05	0.98–1.13	0.142			
Have notification forms	0.46	0.22–0.95	0.037*	0.30	0.03–2.96	0.285
Number of patients seen per day	1.00	1.00–1.01	0.578			
Workload prevents notification	0.83	0.76–0.91	<0.001*	0.84	0.70–0.99	0.043*
Feedback given	0.92	0.82–1.03	0.151			
Data not used for response	0.88	0.78–0.99	0.029*	0.84	0.71–0.99	0.040*

*P-value significant at 5% level.

95% CI = 95% Confidence Interval. ICU = Intensive Care Unit. NDSS = Notifiable Diseases Surveillance System

HCPs’ willingness to notify, experience, training on the NDSS, understanding of the purpose of NDSS, knowledge of what to notify, and perception of feedback given, had no association with correct notification. Working as infection control nurses was also not associated with the correct notification of communicable diseases.

## Discussion

This is one of the first studies in democratic South Africa to determine HCPs’ compliance with the NDSS at a national level and to determine the factors associated with compliance. The study found that 58% of HCPs indicated that they diagnosed a notifiable disease in the year preceding the survey and that 92% of these indicated that they have reported the disease. However, only 51% of those notified the disease/s correctly to the DOH.

A 2015 empirical study that compared South African notifications and laboratory surveillance, found that only 1.5% of suspected measles and meningococcal meningitis cases were notified [42]. This in sharp contrast to the HCP self-reports of 92% notification. The latter is also in contrast to the finding of a 1985–88 SA study that found that only one in seven hepatitis B cases were notified in South Africa [36]. The variations could be due to differences in the denominator (the record reviews measured reporting per case while our survey does this per HCP), and the social desirability bias of self-reported information. The self-reported timeliness of the notifications (67% within 24 hours) was also high. This was in contrast to the poor perceptions of timeliness among key stakeholders, where 45% of national and provincial stakeholders considered the NDSS as timely [35]. Nonetheless, the high proportion of participants that reported notification, albeit incorrectly, is a reflection of HCP willingness to comply with the NDSS.

In this study, one in two (51%) of HCPs reported correct notification of diseases. A 2002 American review, that measured HCP compliance with TB, HIV and sexually transmitted diseases (STIs) reporting, found it to be 79%, and compliance of 49% when dealing with other diseases [8]. Our study excluded HIV, which is not notifiable in South Africa, and is based on HCPs reporting their compliance. We found a similar level of reported compliance as for other diseases in the American review and a lower level than for TB and STIs. Despite the difficulty of cross-country comparisons, and the methodological difference between this study and the American study, our study finding suggests low compliance, particularly in light of South Africa’s high TB disease burden and the fact that TB is the most common notifiable disease [43]. The reported compliance in our study is also lower than a 2014 Irish study which found 98% compliance, when hospital data was compared to notifications [33], and a Taiwan study which found 83.5% compliance amongst HCPs [23]. In Africa, a Nigerian state study found that only 38.2% of HCPs were aware of notifications and 71% reported disease notification [29]. A 2008 Nigerian study in six cities found that 66.5% of doctors reported disease notification, with 65% of those that did not notify indicating that they never diagnosed a notifiable disease [31]. Differences with our study could be because we only focused on notification practices in the preceding year. However, a NDSS cannot function effectively when only half of HCPs notify diseases correctly, particularly when it is not mandatory for laboratories to notify diseases.

We found that significantly fewer HCPs in the private sector diagnosed notifiable diseases. This could be because the private sector serves people with private health insurance, and communities who are dependent on the public sector have a higher incidence of communicable diseases [44]. The study found that a significantly higher number of public sector nurses diagnose and report a notifiable disease. This is because PHC facilities in the public sector are staffed primarily by nurses, who play a greater role in the diagnosis and management of diseases [44]. This study also found that private sector nurses were less likely than those in the public sector nurses to notify correctly, even though they reported the highest level of training in the NDSS. Hence, they should be a focus group for NDSS intervention programmes. Our study found that similar proportions of public and private sector doctors diagnosed or notified diseases. These findings differ from the findings of other studies that reported differences between the two groups. An Indian study [27] and a study in Portugal [11] showed higher levels of compliance amongst public sector doctors, while a Maltese study showed earlier reporting amongst private doctors [13]. The implication of our finding is that similar interventions could be applied amongst doctors in both sectors to improve compliance with the NDSS. The finding that private HCPs in the peripheral and rural province of Limpopo notified fewer diseases correctly indicates the need for interventions to target rural areas. The 50% of private sector doctors (who were mostly GPs) who reported correct notification was comparable to the findings of a 2007 study which showed notification compliance of 37% amongst GPs in Gauteng province [38].

Although there was a statistical association between HCPs perceptions of workload and their reported compliance, there was no statistically significant association between compliance and the reported number of patient consultations per day. This could be because workload is influenced by disease burden, patient management, the number of patients and time spent per patient. However, these factors were not assessed in our study. Our study further showed that HCPs opinions on the usefulness of notification were associated with correct notification to the DOH. This implies that the DOH should communicate to HCPs that notifications influence outbreak management and response. Our study found willingness to notify, training on the NDSS, understanding of the purpose of the NDSS, knowledge on what to notify, possession of notification forms and feedback did not influence HCP compliance with the NDSS. Other studies have found these factors to be important [8, 19, 20, 25, 28, 32, 37–39]. The differences in our findings and these studies may relate to differences in methodology, and do not mean that these are not important factors that require ongoing attention in ensuring an effective NDSS. Our finding that a high percentage of HCPs did not notify correctly indicates that clear guidelines are needed to ensure optimal functioning of the South African NDSS.

Most HCPs indicated that they reported the diagnosed notifiable disease to other HCPs, probably with the expectation that those individuals would report the disease to the DOH. Infection control nurses were the group most diseases were reported to. However, the study found that being an infection control nurse was not associated with correct notification.

A limitation of the study is the self-reported information that may have been influenced by social desirability bias. Another limitation is its cross-sectional nature—it provides a picture of practices at the time of the study. With the high clinical staff turn-over in South Africa [45], practices are unlikely to remain static and ongoing evaluations are needed. The survey also did not allow HCPs who diagnosed more than one notifiable disease in the preceding year, to differentiate their notification practices for these diseases. This might lead to an over-estimation of compliance among HCPs.

Despite the above limitations, the study has provided valuable insights into reported compliance with the NDSS in SA and showed that 51% of study participants reported correct notification, which we have used as proxy for compliance with the NDSS. HCPs demonstrated a willingness to participate with the NDSS when the percentage who reported diseases incorrectly to other HCPs is also taken into consideration. This level of willingness is encouraging and provides a foundation for the introduction of NDSS reforms. These include the introduction of a simplified notification system that makes it easy for clinicians to notify diseases to the correct authority, thus enabling the implementation of appropriate public health measures. Given the high penetration of mobile technology in the country and successful pilot studies on malaria surveillance with mobile technology [46], this is an area that should be explored in addressing the performance of the NDSS as part of the South African health sector reforms. Changes in the NDSS should be accompanied with clearly communicated guidelines and support programmes, both for the public and private health sectors. In other countries, infection control nurses played a critical role in improving communicable disease reporting [47, 48]. In our study, a high percentage of HCPs reported notifiable diseases to them, hence infection control nurses should be used more effectively as a channel of communication between the DOH and HCPs. Paediatricians should also be a specific focus group for training on NDSS and other appropriate interventions, considering their lower compliance with the NDSS and their work with children, who are prone to childhood infections.

## Conclusions

Although a high percentage of HCPs reported compliance with notification of diseases, this compliance was not according to prescribed standards. Hence, the compliance of HCPs in South Africa with the NDSS is suboptimal. The study found that HCPs perceptions of workload and usefulness of notifications were associated with their compliance with the NDSS. In light of the important role of HCPs in the effective functioning of the NDSS, regular feedback to HCPs on the usefulness of notifications, training of paediatricians and rural private sector doctors, as well as guidelines on correct notification procedures are needed to increase their compliance in South Africa.

## Supporting information

S1 File Health care providers’ questionnaire. (DOCX)
