# Supplementary material for: Health care providers’ compliance with the notifiable diseases surveillance system in South Africa
Source: PLoS One. 2018 Apr 9;13(4):e0195194. doi: 10.1371/journal.pone.0195194 (PMC5891014; doi:10.1371/journal.pone.0195194)
Supplement: S1 File — (DOCX) [file pone.0195194.s001.docx]

## S1 File. Health Care Providers’ Questionnaire

**Analysing the National Notifiable Diseases Surveillance System in South Africa**

**For Official use only**

|  | **Questionnaire serial number** |  | \|  \|  \|  \|  \| \| --- \| --- \| --- \| --- \| |
| --- | --- | --- | --- | --- | --- | --- | --- |
| 1. 2 | **Province ID** |  | \|  \|  \|  \| \| --- \| --- \| --- \| |
|  | **District ID** |  | \|  \|  \|  \| \| --- \| --- \| --- \| |
|  | **Sector** | 🞎 Public 1  🞎 Private 2 | \|  \| \| --- \| |
|  | **Health Facility ID** |  | \|  \|  \|  \| \| --- \| --- \| --- \| |
| 1. **8** | Date of survey: | **DD/MM/YY** | \|  \|  \|  \|  \|  \|  \| \| --- \| --- \| --- \| --- \| --- \| --- \| |
| 1. 12 | **Was the questionnaire completed?** | 🞎 No 0  🞎 Yes 1 | \|  \| \| --- \| |
| 1. **15** | Date reviewed: | **DD/MM/YY** | \|  \|  \|  \|  \|  \|  \| \| --- \| --- \| --- \| --- \| --- \| --- \| |

**Please insert the completed questionnaire in the box provided.**

**Consent to Participate**

I have been given an information sheet and I understand the objectives of the study. I further understand that my responses will be kept confidential and that it is up to me whether or not to complete the questionnaire. It has been explained to me that even if I choose not to complete this questionnaire, I should still return it to the box provided and indicate No in the space below. My refusal to participate will in no way prejudice me. I agree voluntarily to complete the questionnaire and to complete the survey only once **(please tick).**

Yes
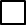
 No
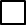


Initial : ………………………………………. Date: ………………………………….

**Section A – Demographic Information**

|  | **Question** | **Official use** |
| --- | --- | --- |
|  | In what professional category do you fall?   - Professional Nurse...1……go to question 2 - Doctor... 2…..go to question 3 |  |
|  | If a Professional Nurse, are you :   - Primary Health Care Trained Nurse...1 - Infection Prevention and Control Practitioner... 2 - Paediatric Trained Nurse...3 - Other... 4 Please specify............................................................................................... |  |
|  | If a doctor, are you :   - Intern...1 - Medical Officer ... 2 - Private General Practitioner ...3 - Registrar... 4 - Infection Prevention and Specialist.... 5 - Family Medicine Specialist.... 6 - Specialist Physician... 7 - Paediatrician... 8 - Other… 9   Please specify……......................…...……………………………………………… |  |
|  | Your age in years ................. |  |
|  | What is your gender?   - Female… 1 - Male… 2 |  |
|  | In which sector are you employed?   - Private… 1 - Public… 2 |  |
|  | In what type of facility do you work?   - Central Hospital...1 - Provincial Tertiary Hospital...2 - Regional Hospital ... 3 - District Hospital... 4 - Private Hospital.... 5 - Community Health Centre... 6 - Clinic... 7 - Private GP practice... 8 - Other... 9 Specify: ................................................................................................... |  |
|  | Did you ever receive training on Notifiable Communicable Diseases?  No Yes ... 1  No No ... 2…………...go to Q11  No Not certain ... 3 .....go to Q11 |  |
|  | What was the duration of the training?  ..................................................................... |  |
|  | How long ago did you receive this training? (In years and months)  ...................................... |  |
|  | Did you receive any formal training in Epidemiology or Surveillance?   - Yes… 1 - No… 2 (If no go to question 14) |  |
|  | If yes, what level of formal training did you receive?   - Certificate… 1 - Diploma… 2 - Bachelor degree… 3 - Master’s degree… 4 - Doctorate… 5 - Other… 6 Please specify…………………….............................................……… |  |
|  | When did you receive your formal training? (Date- year)  ................................................................................................................................................ |  |
|  | How many years of experience do you have working with Notifiable Diseases? (answer 0 if less than one year)  ................................................................................................................................................. |  |

**Section B – Practices related to the Notifiable Diseases Surveillance System**

|  | **Question** | **Official use** |
| --- | --- | --- |
|  | Have you diagnosed any notifiable infectious diseases in the last year?   - Yes...1 (please answer all questions below) - No... 2 (please go to question 20 and answer all questions after that) - Unsure... 3 (please go to question 21 and answer all questions after that) |  |
|  | If yes, which notifiable infectious diseases did you diagnose?  ………………………………………………………………………………….…………..............................................  ................................................................................................................................................ |  |
|  | Did you notify the infectious disease(s)?   - Yes… 1 - No… 2 - Unsure... 3 |  |
|  | Who did you notify the disease(s) to?  ……………………………….............................................……………...........................……. |  |
|  | How long after diagnosis did you notify the disease?   - Within 24 hours… 1 - Within 48 hours… 2 - Within 72 hours... 3 - Within 1 week... 4 - Within 2 weeks... 5 - Longer than 2 weeks... 6 |  |
|  | If you did not notify the disease(s) diagnosed, please state the reasons for not notifying:  ................................................................................................................................................  ................................................................................................................................................ |  |
|  | How many patients do you attend to personally per day?  ................................................................................................................................................... |  |
|  | Do you have notification forms available in your facility/practice?   - Yes… 1 - No… 2 - Unsure... 3 |  |

**Section C –Knowledge and Skills on the Notifiable Diseases Surveillance System (NDSS)**

Below is a list of skills that you need and use as part of your participation in the NDSS. For each, please indicate what your current level of skill is to perform the task on a scale of 1-10 ( with 1 being Low skills, needing more support or training) and 10 being (Very high skills, no support or training needed). Use X to mark the relevant block

|  | **NOTIFICATION** | **MY SKILLS**  **Low Skills** | | | | | **Very High Skills** | | | | | **Office**  **Use** |
| --- | --- | --- | --- | --- | --- | --- | --- | --- | --- | --- | --- | --- |
|  | I know and understand the need for a notifiable diseases surveillance system | 1 | 2 | 3 | 4 | 5 | 6 | 7 | 8 | 9 | 10 |  |
|  | I know which diseases should be notified immediately on clinical suspicion | 1 | 2 | 3 | 4 | 5 | 6 | 7 | 8 | 9 | 10 |  |
|  | I know which diseases should be notified within 24 hours of laboratory confirmation of diagnosis | 1 | 2 | 3 | 4 | 5 | 6 | 7 | 8 | 9 | 10 |  |
|  | I know which diseases can be notified after 24 -48 hours after laboratory confirmation of diagnosis | 1 | 2 | 3 | 4 | 5 | 6 | 7 | 8 | 9 | 10 |  |
|  | I know what process to follow in notifying a disease | 1 | 2 | 3 | 4 | 5 | 6 | 7 | 8 | 9 | 10 |  |
|  | I am able to train other team members on the notification of diseases | 1 | 2 | 3 | 4 | 5 | 6 | 7 | 8 | 9 | 10 |  |
|  | **CASE MANAGEMENT** | **Low Skills** | | | | | **Very High Skills** | | | | |  |
|  | I am confident in the management of meningococcal meningitis | 1 | 2 | 3 | 4 | 5 | 6 | 7 | 8 | 9 | 10 |  |
|  | I am confident in the management of measles | 1 | 2 | 3 | 4 | 5 | 6 | 7 | 8 | 9 | 10 |  |
|  | I am confident in the management of typhoid | 1 | 2 | 3 | 4 | 5 | 6 | 7 | 8 | 9 | 10 |  |
|  | I am able to access the latest protocols and guidelines on notifiable diseases | 1 | 2 | 3 | 4 | 5 | 6 | 7 | 8 | 9 | 10 |  |
|  | I know who to consult if I am uncertain on the management of any notifiable disease | 1 | 2 | 3 | 4 | 5 | 6 | 7 | 8 | 9 | 10 |  |
|  | I am able to train other team members on the management of meningococcal meningitis | 1 | 2 | 3 | 4 | 5 | 6 | 7 | 8 | 9 | 10 |  |
|  | I am able to train other team members on the management of measles | 1 | 2 | 3 | 4 | 5 | 6 | 7 | 8 | 9 | 10 |  |
|  | I am able to train other team members on the management of typhoid | 1 | 2 | 3 | 4 | 5 | 6 | 7 | 8 | 9 | 10 |  |
|  | **PREVENTION AND TRAINING** | **Low Skills** | | | | | **Very High Skills** | | | | |  |
|  | I am knowledgeable on the prevention of notifiable diseases | 1 | 2 | 3 | 4 | 5 | 6 | 7 | 8 | 9 | 10 |  |
|  | I am able to educate my patients on the prevention of notifiable diseases | 1 | 2 | 3 | 4 | 5 | 6 | 7 | 8 | 9 | 10 |  |
|  | I provide access for my patients to education material on the prevention of notifiable diseases in my facility | 1 | 2 | 3 | 4 | 5 | 6 | 7 | 8 | 9 | 10 |  |
|  | I am able to train other team members on the prevention of communicable diseases | 1 | 2 | 3 | 4 | 5 | 6 | 7 | 8 | 9 | 10 |  |

**Section D- Perceptions on the Notifiable Diseases Surveillance System (NDSS)**

| Listed below are statements on Attributes of the Notifiable Disease Surveillance. Using the provided scale state how strongly you agree or disagree with the statement.  **PLEASE ANSWER ALL QUESTIONS – DO NOT LEAVE ANY OUT.** | | | | | | | | |
| --- | --- | --- | --- | --- | --- | --- | --- | --- |
|  | Statement | Strongly Disagree | Disagree | Disagree slightly | Neither agree or disagree | Agree Slightly | Agree | Strongly agree |
|  | The form used to notify diseases is easy to understand | 1 | 2 | 3 | 4 | 5 | 6 | 7 |
|  | The form used to notify diseases takes a long time to fill in | 1 | 2 | 3 | 4 | 5 | 6 | 7 |
|  | The notification process is not easy to comply with | 1 | 2 | 3 | 4 | 5 | 6 | 7 |
|  | Meningococcal meningitis does not need to be notified within 24 hours of clinical suspicion | 1 | 2 | 3 | 4 | 5 | 6 | 7 |
|  | Measles must be notified within 24 hours of diagnosis | 1 | 2 | 3 | 4 | 5 | 6 | 7 |
|  | Typhoid can be notified after 48 hours of diagnosis | 1 | 2 | 3 | 4 | 5 | 6 | 7 |
|  | I am willing to participate in the notifiable disease surveillance system | 1 | 2 | 3 | 4 | 5 | 6 | 7 |
|  | Data obtained through the notifiable disease surveillance system is not used for outbreak response | 1 | 2 | 3 | 4 | 5 | 6 | 7 |
|  | Outbreak response teams do respond timely to most outbreaks | 1 | 2 | 3 | 4 | 5 | 6 | 7 |
|  | Data obtained through the notifiable disease surveillance system is used for policy and guideline formulation | 1 | 2 | 3 | 4 | 5 | 6 | 7 |
|  | Data obtained through the notifiable disease surveillance system do not contribute to knowledge on the prevention and control of infectious diseases | 1 | 2 | 3 | 4 | 5 | 6 | 7 |
|  | The notifiable disease surveillance system has been changed to meet changing circumstances and needs in the last three years | 1 | 2 | 3 | 4 | 5 | 6 | 7 |
|  | Lack of facility supervision do not impact on compliance with the system | 1 | 2 | 3 | 4 | 5 | 6 | 7 |
|  | The department provided no feedback to providers on notifiable diseases over the last year | 1 | 2 | 3 | 4 | 5 | 6 | 7 |
|  | A high workload prevents me from notifying diseases | 1 | 2 | 3 | 4 | 5 | 6 | 7 |
|  | A lack of access to communication equipment prevents me from notifying diseases | 1 | 2 | 3 | 4 | 5 | 6 | 7 |

**Section E – Other Comments on the Notifiable Diseases Surveillance System**

1. **Please rate the availability of staffing for the notifiable diseases surveillance system at the following levels:**

|  | Organisational level | Very Poor | Poor | Satisfactory | Good | Very Good |
| --- | --- | --- | --- | --- | --- | --- |
|  | National | 1 | 2 | 3 | 4 | 5 |
|  | Province | 1 | 2 | 3 | 4 | 5 |
|  | District | 1 | 2 | 3 | 4 | 5 |
|  | Facility | 1 | 2 | 3 | 4 | 5 |

1. **Please rate the level of investment of funding for the notifiable diseases surveillance system at the following organisational levels:**

|  | Organisational level | Very Poor | Poor | Satisfactory | Good | Very Good |
| --- | --- | --- | --- | --- | --- | --- |
|  | National | 1 | 2 | 3 | 4 | 5 |
|  | Province | 1 | 2 | 3 | 4 | 5 |
|  | District | 1 | 2 | 3 | 4 | 5 |
|  | Facility | 1 | 2 | 3 | 4 | 5 |

1. **Please rate the organisational capacity for the notifiable diseases surveillance system at the following levels:**

|  | Organisational level | Very Poor | Poor | Satisfactory | Good | Very Good |
| --- | --- | --- | --- | --- | --- | --- |
|  | National | 1 | 2 | 3 | 4 | 5 |
|  | Province | 1 | 2 | 3 | 4 | 5 |
|  | District | 1 | 2 | 3 | 4 | 5 |
|  | Facility | 1 | 2 | 3 | 4 | 5 |

1. **Please indicate to what extent the following interventions would benefit the notifiable diseases surveillance system (on a scale of 1 to 10, with 1= No benefit and 10 = Maximum benefit)**

|  | | Intervention | No benefit | | | | | Maximum benefit | | | | | |  |
| --- | --- | --- | --- | --- | --- | --- | --- | --- | --- | --- | --- | --- | --- | --- |
|  | | Addressing staffing gaps | 1 | 2 | 3 | 4 | 5 | 6 | 7 | 8 | 9 | | 10 |  |
|  | | Addressing the gaps in the organisational capacity of the department | 1 | 2 | 3 | 4 | 5 | 6 | 7 | 8 | 9 | | 10 |  |
|  | | Investing more financial resources in the system | 1 | 2 | 3 | 4 | 5 | 6 | 7 | 8 | 9 | | 10 |  |
|  | | Introduction of the use of an electronic system | 1 | 2 | 3 | 4 | 5 | 6 | 7 | 8 | 9 | | 10 |  |
|  | | Introduction of mobile technology | 1 | 2 | 3 | 4 | 5 | 6 | 7 | 8 | 9 | | 10 |  |
|  | Do you have any further comments on the Notifiable Diseases Surveillance System  .......................................................................................................................................  .......................................................................................................................................  .......................................................................................................................................  ....................................................................................................................................... | | | | | | | | | | |  | | |
|  | Do you have any recommendations on how to improve the Notifiable Diseases Surveillance System  .......................................................................................................................................  .......................................................................................................................................  .......................................................................................................................................  ....................................................................................................................................... | | | | | | | | | | |  | | |

**Thank you for participating**
